# Mental health legislation in Botswana

**DOI:** 10.1192/bji.2018.24

**Published:** 2019-08

**Authors:** J. Maphisa Maphisa

**Affiliations:** Lecturer, Department of Psychology, University of Botswana, Private Bag, 00705 Gaborone, Botswana. Email: maphisa.maphisa@mopipi.ub.bw

**Keywords:** Human rights, psychiatry and law, stigma and discrimination

## Abstract

The Mental Disorders Act of 1969 is the primary legislation relating to mental health in Botswana. Despite the country not being a signatory to the United Nations Convention on the Rights of Persons with Disabilities, its Act has a self-rated score of four out of five on compliance to human rights covenants. However, it can be argued that the Act does not adequately espouse a human rights- and patient-centred approach to legislation. It is hoped that ongoing efforts to revise the Act will address the limitations discussed in this article.

## Country context

Botswana is a landlocked, upper-middle-income country (World Bank, 2015) in southern Africa with a population of two million people (Statistics Botswana, 2011). The country has a ratio of 17.7 mental health practitioners per 100 000, the majority being nurses (WHO, 2014). There are 0.29 psychiatrists and 0.37 and psychologists per 100 000 people in Botswana. There is only one psychiatric hospital, with 300 beds (Statistics Botswana, 2017), located 80 km from the capital city, Gaborone. There are five psychiatric units in general hospitals across the country, and about 390 psychiatric beds in the whole country (Sidandi *et al*., 2011).

## Objective of the act

The Mental Disorders Act of 1969 (CAP. 63:02, Laws of Botswana enacted in 1971, hereinafter Act) was formulated to ‘… make provision for the reception, detention,^1^ treatment and protection of mentally disordered persons’. The Act is mainly procedural and is not protectionist with respect to the rights of individuals with mental disorders or defects. The Act does not offer any protection to persons with mental disorders outside the processes of reception and detention. Furthermore, the Act does not explicitly mandate the care and treatment of persons with mental disorders once received or detained.

## Types of patients

The Act has three categories of patients. Class I patients are those who pose a risk to themselves (e.g. suicidal) or others (e.g. homicidal). Class II patients are those who are vulnerable to abuse, cannot look after themselves, and require skilled medical attention. Class III patients are similar to Class II patients, but differ in that they do not require skilled medical attention.

## Involuntary detention procedure

Parts II to VI of the Act mainly describe procedures to be taken in the reception and detention of Class I and Class II patients. Although Class III patients may be covered by these procedures, the Act tends to treat these patients via a separate dispensation (see Private Dwelling Patients below).

Involuntary detention may occur through either of two processes, which do not consider informed consent by, or assent from, the patients. These two processes at some point require the involvement of a health facility Superintendent, a District Commissioner (DC), a Master of the High Court (hereinafter Master) and a Director of Medical Services (hereinafter Director). The DC is the head of a district and serves as a central government representative in the district (Sharma, 2010).

### Process 1: Reception order

A family member or any other person who has attained the age of 21 years who has seen the patient within the preceding 14 days of the application can apply to the DC for a reception order. The reception order authorises ‘the patient to be removed to, and to be received and detained in, an institution or place of detention to be named in the order’. The applicant has to state why they believe that the family member is mentally disordered or defective. It is not required that the applicant state that the patient needs treatment. Upon receipt of the application, the DC must inquire on the matter publicly or privately, and obtain an opinion from a medical practitioner on the patient's mental state. The DC may also visit the patient, or order that the patient be brought before a medical practitioner or himself if the patient will not voluntarily appear. The DC has discretion in accepting or denying the reception order application, but is bound by the opinion of the medical practitioner in determining to which class the patient belongs. If satisfied, the DC issues a reception order prescribing the duration and institution for detention or temporary treatment. The detention cannot be for more than 30 days, but it may be extended by the DC up to a maximum cumulative detention period of 90 days. In issuing the reception order, the DC sends a copy to the Master, and provides an inventory of the patient's assets. Upon admission to the health establishment, the Superintendent within 10 days remits a report of the patient's mental state to the Master, who may order further detention, appoint a *curator bonis*, or discharge the patient if requested by the Superintendent. The Master's order for further detention may be ‘either for a definite or for an indefinite period’.

### Process 2: Urgent Application

The urgent application seems to envisage a scenario of a patient experiencing an acute episode of psychopathology where danger to the patient or society is imminent, and where the involvement of the DC's inquiry to issue a reception order would impede the expedient delivery of care to the patient. A family member or any other person who has attained the age of 21 years and who has seen the patient in the preceding 48 hours makes an application to a health establishment. The applicant needs to state why the patient is mentally disordered or defective, and must produce a medical certificate based on an examination carried out within the preceding 48 hours. Upon admission of the patient to the health facility, the applicant of the urgent application must immediately report the matter to the DC for an application of a reception order. This reception order should be granted within 14 days; if not, the patient is released. The DC can order further detention (not more than 14 days or cumulative 28 days from reception at the health facility), reject the reception order application or issue a reception order. If the reception order is granted, an identical process detailed in Process 1 above ensues. If the reception order is not granted, it would be unlawful to detain the patient further.

The Act makes no provision for assisted admissions where the patient is incapable of making decisions but is willing to receive treatment. Patients under these circumstances are dealt with as involuntary patients.

The Act provides three ways in which detention may be terminated. Termination of detention can be instituted upon expiration of the duration stated in the reception order or urgent application, or upon a successful appeal of the detention to the High Court. Furthermore, patients are released upon the cessation of an active phase of psychopathology. In this latter instance, the termination of detention requires certification by two medical practitioners. Following the certification, the release is ordered by the Master as per the Director's recommendations.

## Voluntary admissions

Part IX of the Act describes procedures for voluntary admissions. The Act does not require that voluntary patients be classified into the three classes discussed above.

Persons of 16 years of age and above may make an application to be ‘received and detained’ as voluntary patients in a health establishment. Those younger than 16 years of age require their parents or guardians to make the application. The decision to ‘receive and detain’ them is at the Superintendent's discretion. Patients are required to give one week's written notice if they wish to discharge themselves from the institution, albeit the Superintendent may allow discharge before the expiration of the notice.

Voluntary patients who, during their admission, by reason of mental illness become incapable of making informed decisions regarding their care are discharged after 28 days from the day they became incapable. Only post-discharge can there be applications for a reception order or urgent application. There is no immediate process to allow for their change to involuntary patient status for purposes of treatment. Thus, it seems that the admitting health establishment is not empowered to provide care and treatment if such an incapable patient refuses the care and treatment.

## Private dwelling patients

The Act allows for the care and maintenance of mentally ill persons (mostly Class III patients) in private dwellings. This allowance may be granted in terms of Section 13 by an order in lieu of a reception order or in terms of an application by a caregiver as described in Section 29. Section 29 relates to individuals whose mental illnesses have continued for 6 months and who require compulsory confinement or restraint to receive care in a private dwelling. Persons with charge of such patients must furnish the DC with a medical practitioner's report detailing the mental and physical condition of the patient, and the reasons private care is desired. The DC shall inform the Director and Master. The Master may grant the request for private care, or recommend an application for a reception order, and appoint a *curator bonis*. The DC is then responsible for monitoring such a patient's care. The care offered in terms of Section 29 is provided by non-remunerated individuals and is provided in a private dwelling.

## Protection of patients

The Act provides some protection for detained persons with mental illness. Orders made regarding them may be appealed and reviewed. Their property is protected via a Master-appointed *curator bonis.* Patients are protected from ill-treatment by practitioners employed in their place of detention. This latter protection includes the limiting of mechanical means of bodily restraint.

A Mental Health Board is prescribed in the Act as an oversight mechanism to ensure that the well-being of detained patients is upheld, that places of detention are conducive and that patients’ complaints are investigated. The Board, of no less than three members, is required to visit health facilities detaining persons with mental illness at least once in 6 months. The Board is required to observe or give audience to every patient.

## Conclusion

The Mental Disorders Act is restricted in focus to the reception and detention of people with mental disorders. The Act neglects to explicitly mandate health facilities with the treatment and care of patients. Furthermore, the Act lacks a robust human rights-oriented and patient-centred approach. An amendment of the Act is called for to align the Act with these contemporary values. The rights of persons with mental illness need to be explicitly articulated in legislation and should be extended beyond the period of ‘detention’.

## Footnotes

1 The use of this word and its variants is in conformity with the Act. However, it is the author's opinion that such terminology is prejudicial and perhaps reflects an underlying stigma in which people with mental illness are detained as if they were dangerous criminals instead of being treated as patients with an illness. This observation has also been made regarding other Acts in the country (see Maphisa, 2016), for example, in the use of the label ‘criminal lunatic’ to describe those not guilty by reason of mental illness.
